# Distinct acute and chronic brain health phenotypes among people living with HIV in a Ugandan clinical cohort: a comorbidity network analysis

**DOI:** 10.21203/rs.3.rs-10497168/v1

**Published:** 2026-07-31

**Authors:** Joseph Mwaka, Raha M. Dastgheyb, Conrad Sserunjogi, Jesca Nataliya, Ronald Mulebeke, Yvonne Karamagi, Leah H. Rubin

**Affiliations:** Mildmay Research Centre Uganda; Johns Hopkins University; Mildmay Research Centre Uganda; Mildmay Research Centre Uganda; Mildmay Research Centre Uganda; Mildmay Research Centre Uganda; Johns Hopkins University

**Keywords:** HIV, brain health, multimorbidity, neurocognitive impairment, ICD-10, administrative data, comorbidity, sub-Saharan Africa, Uganda, latent class analysis

## Abstract

**Background.:**

Individual brain health conditions among people living with HIV in sub-Saharan Africa are increasingly well documented, but the co-occurrence of comorbidity and multimorbidity across distinct neurological and psychiatric conditions remains poorly characterized in this setting. We examined the co-occurrence structure of brain health conditions in a specialist HIV clinical cohort in Uganda.

**Methods.:**

We analyzed 1,137 ICD-10-coded records from 581 patients in a specialist HIV neuropsychiatric cohort in Kampala (2017 to April 2026). All ICD-10 codes were classified, based on expert clinical consultation, into eight brain health domains and a two-level phenotype (acute versus chronic). Pairwise cooccurrence among domains with at least ten patients was tested using phi coefficients with bootstrap confidence intervals and Benjamini-Hochberg correction; latent class analysis was a pre-specified sensitivity analysis.

**Results.:**

Of 573 classifiable patients, 476 (83.1%) carried a single brain health domain and only 12 (2.1%) carried diagnoses from both the acute and chronic phenotypes. Depression, bipolar and psychosis spectra co-aggregated (phi 0.11 to 0.25), while opportunistic CNS infection was mutually exclusive with every chronic domain (phi as low as −0.734). Cognitive impairment showed no significant association with major depressive disorder (12 patients with both domains; phi − 0.064, q = 0.203). Latent class analysis independently recovered a multimorbid chronic phenotype (24.6% of patients).

**Conclusions.:**

Brain health conditions in this cohort partition into two clearly separated phenotypes: an acute opportunistic-infection phenotype and a chronic phenotype in which depressive, bipolar and psychotic presentations co-aggregate. Cognitive impairment and major depressive disorder, a comorbidity widely assumed to be common in HIV, showed no significant association in this cohort. Precise, clinically grounded classification of ICD-10 diagnoses is important for valid comorbidity research in HIV neuropsychiatry.

## Background

People living with HIV experience brain health conditions arising from at least two distinct pathophysiological processes, and whether these processes form separable clinical phenotypes or an undifferentiated multimorbid burden within the same patients has not been evaluated in sub-Saharan Africa. Opportunistic central nervous system infections, principally cryptococcal meningitis, are complications of severe immunosuppression rather than direct consequences of HIV neuropathogenesis; they occur predominantly at CD4 counts below 200 cells/μL and cluster around advanced disease presentation or antiretroviral therapy initiation, accounting for an estimated 19% of AIDS-related deaths globally[1]. HIV-associated neurocognitive impairment follows a different course: it is attributed to sustained neuroinflammation over years of infection and typically accumulates well into a patient’s treatment course rather than at presentation[2]. Depression, bipolar and psychotic spectrum disorders represent a further set of conditions with distinct biological and psychosocial drivers. These conditions therefore differ in mechanism and in the point of the disease course at which they typically arise, and it is not established whether they cluster together or occupy separable phenotypes in the same population.

Existing regional evidence addresses each condition individually. HIV-associated neurocognitive impairment shows wide reported variation: a meta-analysis of 49 African studies found a pooled prevalence of 46%, with individual study estimates ranging from 14 to 88% depending on the screening tools and normative standards used[3]; illustrating this sensitivity, a Ugandan clinic-based study using a brief bedside screening instrument reported probable HIV dementia in 64.4% of attendees[4]. Depression is the most extensively studied condition in this population: an earlier sub-Saharan African synthesis reported treated-population estimates ranging from 9 to 32% depending on the measurement instrument used[5], and a more recent meta-analysis of 34 studies and 21,143 adults on antiretroviral therapy across Africa estimated a pooled prevalence of 36% (95% confidence interval 27 to 40%)[6]. Bipolar and psychotic spectrum disorders remain comparatively understudied; clinic-based data from urban Uganda documented bipolar affective disorder in 17.4% of HIV-positive attendees, a prevalence substantially exceeding general-population estimates in the same setting[7].

A more fundamental gap runs through this literature: none of it examines how these conditions co-occur within the same patients. Prevalence estimates reported one condition at a time cannot show whether two conditions cluster together, occur independently, or are mutually exclusive in the same population, a distinction with direct consequences for how screening and care should be integrated. The handful of studies that do report overlap take diagnostic category labels at face value, without asking whether a category such as HAND/HIV-CNS captures a single coherent clinical construct. This matters in HIV neuropsychiatry specifically: a substantial proportion of diagnoses coded as cognitive impairment are in practice secondary psychiatric syndromes attributed to a physiological cause, and the diagnostic criteria used for HIV-associated neurocognitive disorders are themselves documented to produce high false-positive rates [8, 9]. A category built from administrative codes can therefore appear to overlap with a psychiatric category for a trivial reason: both labels were applied to the same clinical presentation, not because the underlying conditions genuinely co-occur.

This study is designed to close both gaps using nearly a decade of routinely collected clinical data from a specialist HIV neuropsychiatric cohort in Kampala. We reclassified every constituent ICD-10 code recorded in the cohort into clinically coherent brain health domains, based on expert clinical consultation rather than administrative groupings, and reported the complete classification scheme in full for transparency and reuse. Using this classification, we tested the pairwise co-occurrence of domains against statistical independence and evaluated whether a multimorbid phenotype can be recovered independently by two separate analytic methods. This approach demonstrated that a clinically grounded classification is necessary before comorbidity between HIV-associated cognitive impairment and depression, two of the most frequently discussed brain conditions in this population, can be reported without risk that the association is an artifact of overlapping diagnostic labels rather than a genuine clinical co-occurrence.

## Methods

### Study design and setting

**Figure 1:**
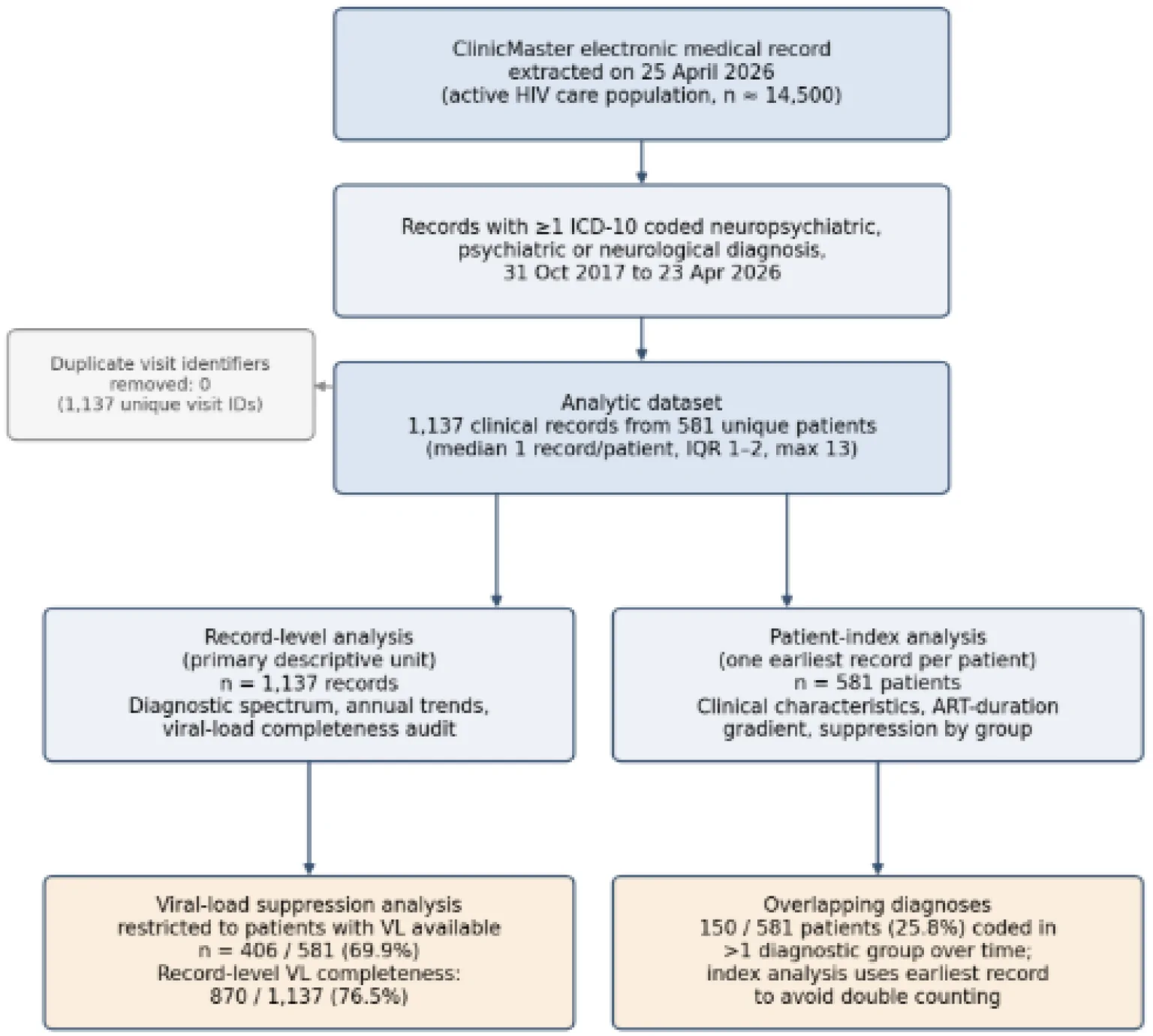
Derivation of the analytic dataset (STROBE/RECORD flow). Data were extracted from the ClinicMaster electronic medical record on 25 April 2026. The 1,137 records from 581 patients were analysed at two levels: a record level (all records) and a patient-index level (one earliest record per patient).

This was a secondary analysis of routinely collected electronic clinical records from the Mildmay HIV neuropsychiatric cohort, a specialist HIV care population at Mildmay Uganda in Kampala. Records were extracted from the ClinicMaster electronic medical record on 25 April 2026. The source population comprised approximately 14,500 patients in active HIV care. The analytic dataset consisted of all clinical records carrying at least one International Classification of Diseases, Tenth Revision (ICD-10) coded neuropsychiatric, psychiatric or neurological diagnosis recorded between 31 October 2017 and 23 April 2026: 1,137 records from 581 unique patients (Fig. 1). Reporting follows the RECORD extension of the STROBE statement for studies using routinely collected health data.

### Analytic units

Two analytic units were used throughout. Record-level analysis (all 1,137 records) describes diagnostic and service burden and may include repeated records from the same patient. Patient-index analysis uses one earliest record per patient (n = 581) to describe unique patients and avoid double-counting. Domain-level co-occurrence used a patient-ever classification, in which a patient contributed to a domain if any of their records across the observation period carried a diagnosis assigned to that domain.

### Classification of brain health domains

All ICD-10 diagnostic codes present in the cohort (105 distinct codes across 1,137 records) were classified, on the basis of expert clinical consultation, into one of eight brain health domains (cognitive impairment, major depressive disorder, bipolar spectrum disorder, psychosis spectrum, anxiety disorders, trauma- and stressor-related disorders, substance use disorder, and opportunistic CNS infection) and a superordinate phenotype (acute brain injury versus chronic brain health multimorbidity). The cognitive impairment domain was defined to comprise organic or cognitive presentations (delirium, amnestic disorder, encephalopathy, unspecified organic mental disorder, and dementia of all subtypes), consistent with contemporary recommendations that cognitive-impairment categories in HIV should reflect clinical presentation rather than administrative chapter placement [9]. A residual category (other or unclassifiable; 12 records, 1.1% of the cohort) captured nonspecific or non-diagnostic codes and was excluded from the co-occurrence network and latent class analysis. The complete code-level classification scheme, including the rationale for each domain assignment, is provided in the Appendix (Tables A and B). Of the 581 patients, 573 (98.6%) had at least one record assigned to a defined brain health domain; the remaining 8 carried only unclassifiable codes across their entire record history and were excluded from the domain-level analysis.

### Statistical analysis

Descriptive statistics summarised the diagnostic spectrum and the distribution of the number of brain health domains per patient. Pairwise co-occurrence was assessed only for domains with at least ten patients, to avoid unstable estimates from sparse cells. For each domain pair we computed the phi coefficient with a 2,000-replicate bootstrap 95% confidence interval, the observed versus expected joint count under independence, and a Fisher exact test. To control for multiple comparisons across the ten tested pairs, p values were adjusted using the Benjamini-Hochberg false discovery rate procedure, with significance defined at an adjusted (q) value below 0.05 [10]. Community structure was identified from the complete signed adjacency matrix of FDR-significant phi coefficients, positive and negative edges together, using spin-glass community detection with a signed implementation that treats positive edges as attractive and negative edges as repulsive [11]; standard modularity-based algorithms, including Louvain, cannot incorporate negative edge weights, so Louvain community detection restricted to the positive-edge subgraph [12] was retained as a sensitivity comparison. As a pre-specified sensitivity analysis, latent class models with one to five classes were fitted to the binary domain indicators meeting the minimum cell-size threshold, with model selection based on the Bayesian information criterion (BIC). Analyses were conducted in R; a fixed random seed was used for the bootstrap and latent class procedures to ensure reproducibility.

### Ethics

This analysis used de-identified routine clinical records. Ethical approval and the applicable consent arrangements are described in the Declarations.

## Results

### Cohort and brain health multimorbidity burden

Classification of the 1,137 records into eight brain health domains showed that 573 of the 581 patients (98.6%) had at least one record assigned to a defined domain. By lifetime (“ever”) classification, major depressive disorder was the most prevalent domain (298 patients, 52.0%), followed by opportunistic CNS infection (210, 36.6%), psychosis spectrum (75, 13.1%), bipolar spectrum disorder (70, 12.2%) and cognitive impairment (31, 5.4%). Anxiety disorders (9, 1.6%), substance use disorder (4, 0.7%) and trauma- and stressor-related disorders (3, 0.5%) were each carried by fewer than ten patients and were therefore excluded from inferential pairwise testing.

Most patients carried a single brain health domain: 476 of 573 (83.1%) had one lifetime domain, 74 (12.9%) had two, 18 (3.1%) had three, and 5 (0.9%) had four or more. Overlap across the phenotype boundary was rare. Only 12 patients (2.1%) carried diagnoses from both the acute brain injury phenotype and the chronic brain health multimorbidity phenotype; 198 (34.6%) carried acute-phenotype diagnoses only and 363 (63.4%) carried chronic-phenotype diagnoses only.

### Pairwise co-occurrence against independence

Ten pairwise associations among the five domains meeting the minimum cell-size threshold were evaluated. Six were significant at the 0.05 false discovery rate threshold (Table 1). Two positive associations, both connecting domains within the chronic phenotype, exceeded chance. The strongest was between psychosis spectrum and bipolar spectrum disorder: 25 patients carried both domains against an expected overlap of 9.2 under independence (observed/expected 2.73; phi 0.250, 95% CI 0.144 to 0.366; q < 0.001). Major depressive disorder co-occurred with bipolar spectrum disorder beyond chance (47 observed versus 36.4 expected; phi 0.113, 95% CI 0.033 to 0.186; q = 0.012). Together these associations connect three of the five domains into a single chronic cluster.

The remaining four significant associations were all negative and all involved opportunistic CNS infection. The strongest mutual exclusion was with major depressive disorder: only 8 patients carried both against an expected overlap of 109.2 (observed/expected 0.07; phi − 0.734, 95% CI − 0.783 to − 0.682; q < 0.001). Opportunistic CNS infection was similarly exclusive of psychosis spectrum (3 versus 27.5 expected; phi − 0.263; q < 0.001), bipolar spectrum disorder (4 versus 25.7; phi − 0.240; q < 0.001) and cognitive impairment (3 versus 11.4; phi − 0.134; q = 0.002). This pattern is consistent with opportunistic CNS infection representing an acute presentation that is clinically and temporally separated from the chronic psychiatric domains.

### Cognitive impairment and major depressive disorder

The cognitive impairment domain (41 records; 31 patients) comprised delirium, amnestic disorder, encephalopathy, unspecified organic mental disorder, and dementia of all subtypes (Appendix, Table B), consistent with recommendations that cognitive-impairment categories in HIV should reflect organic clinical presentation rather than broader administrative chapter placement^[9]^. Only 12 patients carried diagnoses of both cognitive impairment and major depressive disorder, against an expected overlap of 16.1 under independence (observed/expected 0.74; phi − 0.064, 95% CI −0.137 to 0.020; Fisher p = 0.142; q = 0.203). This association was not only non-significant but numerically negative, indicating no evidence of co-aggregation between cognitive impairment and depression in this cohort once cognitive impairment is defined on strictly organic clinical grounds.

### Network community structure

Signed spin-glass community detection, applied to the complete adjacency matrix of FDR-significant phi coefficients with positive and negative edges together, identified two communities (Table 2). Community 1 comprised major depressive disorder, bipolar spectrum disorder, psychosis spectrum and cognitive impairment; community 2 comprised opportunistic CNS infection alone. Cognitive impairment’s membership in community 1 was driven entirely by its negative association with opportunistic CNS infection (phi − 0.134), the only significant edge it carries: cognitive impairment showed no significant positive association with any of the three psychiatric domains (phi − 0.064 to 0.067, all q ≥ 0.16), so this grouping reflects shared exclusion from the acute phenotype rather than genuine co-aggregation with depression, bipolar disorder or psychosis. Louvain community detection restricted to the positive-edge subgraph, which cannot represent this exclusion, instead placed cognitive impairment in an isolated third community (Table 2), while recovering the same chronic cluster (major depressive disorder, bipolar spectrum disorder and psychosis spectrum) and the same acute, opportunistic-infection-only community as the signed method.

### Latent class analysis: sensitivity

The BIC-preferred latent class model identified three classes (BIC 2,172.4; entropy 0.856), an improvement on the two-class (BIC 2,236.3) and four-class (BIC 2,183.1) alternatives (Table 3). Two classes were dominated by a single domain: a depression class (39.6% of patients; conditional probability of major depressive disorder 1.00, all other domains ≤ 0.09) and an opportunistic-infection class (35.8%; conditional probability 1.00, all others ≤ 0.03). The third class (24.6%) loaded meaningfully on multiple chronic domains simultaneously: psychosis spectrum 0.52, major depression 0.47, bipolar spectrum disorder 0.34 and cognitive impairment 0.20, with negligible loading on opportunistic CNS infection (0.03; Table 4). This multimorbid class corresponds closely to the chronic cluster identified independently in the network analysis, and its near-zero loading on opportunistic CNS infection reinforces the acute versus chronic separation. The convergence between two methods (one based on pairwise co-occurrence, the other on latent class structure) strengthens confidence in the two-phenotype architecture.

## Discussion

To our knowledge, this is the first study to test the co-occurrence structure of brain health conditions among people living with HIV in sub-Saharan Africa against statistical independence using a clinically grounded classification of routine ICD-10 diagnoses. Three principal findings emerge. First, the diagnostic space partitioned into two clearly separated phenotypes: an acute phenotype defined by opportunistic CNS infection, mutually exclusive with every chronic domain, and a chronic phenotype in which major depressive disorder, bipolar spectrum disorder and psychosis spectrum co-aggregate. Second, cognitive impairment, when defined on strictly organic clinical grounds, showed no significant association with major depressive disorder, a comorbidity often assumed to be common in HIV. Third, latent class analysis independently recovered a multimorbid chronic phenotype whose profile is dominated by the three chronic psychiatric domains and is essentially unloaded on opportunistic CNS infection.

The mutual exclusion between opportunistic CNS infection and every chronic domain is biologically coherent. Opportunistic CNS infections in this cohort were overwhelmingly cryptococcal (75% of the domain’s records), and cryptococcal meningitis occurs almost exclusively at profound immunosuppression, typically at CD4 counts below 100 cells per microlitre, presenting near diagnosis or antiretroviral therapy initiation and accounting for a large share of AIDS-related mortality in the region [1]. Affective, psychotic and cognitive presentations, by contrast, accumulate over years of treatment [2]. The two groups therefore occupy different points in the HIV treatment trajectory, which plausibly explains their near-complete separation in the same clinical population.

The absence of association between cognitive impairment and major depression in this cohort contrasts with the frequent assumption, elsewhere in the HIV literature, that the two conditions commonly co-occur. This difference may reflect the classification approach used here, in which cognitive impairment was restricted to organic and cognitive presentations (delirium, amnestic disorder, encephalopathy, unspecified organic mental disorder, and dementia) rather than a broader chapter-level grouping. Administrative codes such as those under the ICD-10 chapter for mental disorders due to a known physiological condition capture the clinician’s causal attribution rather than the underlying phenotype, and secondary depressive or psychotic presentations can be coded under the same chapter as organic cognitive syndromes. Because the criteria used to define HIV-associated neurocognitive disorders are already known to carry high false-positive rates and to be sensitive to how impairment is operationalised, precise, clinically grounded classification of constituent diagnoses is important for valid comorbidity research in HIV neuropsychiatry, particularly for a comorbidity as clinically consequential as cognitive impairment and depression [9].

The chronic cluster identified here, centred on the bipolar-psychosis association and connected to major depression through bipolar disorder, is consistent with the transdiagnostic severe mental illness literature, in which the boundary between bipolar and psychotic disorders has long been recognised as porous [13]. That this structure is recovered in an HIV clinical cohort in East Africa using routine ICD-10 codes, rather than structured diagnostic instruments, suggests that the underlying phenotypic architecture persists across clinical contexts. Notably, cognitive impairment did not co-aggregate with any of the three chronic psychiatric domains: all three pairwise associations were non-significant (phi − 0.064 to 0.067). Community detection that incorporates negative as well as positive associations grouped cognitive impairment with the chronic cluster, but only because it shares the same exclusion from opportunistic CNS infection as the psychiatric domains, not because of any positive association with them; community detection restricted to positive edges, unable to represent this exclusion, placed cognitive impairment in an isolated community instead. Defined on strictly organic clinical grounds, cognitive impairment therefore remains phenotypically distinct from the mood-psychosis spectrum in this cohort regardless of which community-detection method is used, and the discrepancy between the two methods is itself informative: it shows that a domain can share a network community with others purely through common exclusions, without any underlying positive co-aggregation, a distinction visible only when negative associations are modelled explicitly rather than discarded.

These findings sit alongside a growing multimorbidity literature in HIV that has, to date, largely been generated in high-income settings and has focused on cardiovascular, metabolic and other non-communicable disease patterns, and alongside data-driven phenotyping of neurocognitive and depressive presentations in well-characterised research cohorts [14, 15]. The present analysis extends this work in two directions: it is set in a routine sub-Saharan African specialist HIV service rather than a research cohort, and it demonstrates the importance of clinically precise diagnostic classification for such phenotyping. If the multimorbid chronic phenotype identified here is confirmed to be older, longer-treated and cognitively vulnerable in a subsequent characterisation analysis incorporating antiretroviral therapy duration, viral suppression and clinical stage, it would define a target population for prospective cognitive screening and integrated mental health care in ageing HIV populations.

### Strengths and limitations

The principal strength of this analysis is a specialist HIV neuropsychiatric dataset spanning nine years combined with a clinically grounded, fully transparent classification of every constituent ICD-10 code. All 105 codes present in the cohort were reviewed individually, with the complete classification scheme and its rationale reported in the Appendix. This transparency supports the finding that cognitive impairment and major depressive disorder are not associated in this cohort once cognitive impairment is defined on strictly organic clinical grounds.

Several limitations require acknowledgement. First, the diagnoses are clinician-coded from routine care rather than derived from structured diagnostic interviews; the absence of a code does not necessarily mean absence of the condition. Second, some domains had small sample sizes: anxiety disorders (9 patients), substance use disorder (4) and trauma- and stressor-related disorders (3) were each too small for pairwise testing, a direct trade-off of diagnostic granularity against statistical power. Cognitive impairment (31 patients) is also a relatively small domain, and its non-significant associations carry wide confidence intervals and should be interpreted as inconclusive rather than as evidence of true independence. Third, CD4 count at antiretroviral therapy start was not available for all patients, precluding a full adjustment for baseline immunological status. Fourth, the cross-sectional lifetime classification does not capture the temporal sequence of diagnoses, and the negative associations between opportunistic infection and chronic domains, while consistent with a treatment-stage interpretation, could also reflect differential survival or referral patterns. Fifth, twelve records (1.1% of the cohort) did not map cleanly onto any brain health domain and were excluded as unclassifiable, and eight patients whose entire record history fell into this residual were excluded from the domain-level analysis.

## Conclusions

Brain health conditions among people living with HIV in routine Ugandan specialist care do not co-occur randomly. The diagnoses partition into an acute opportunistic-infection phenotype that is mutually exclusive with all chronic brain health conditions, and a chronic phenotype in which bipolar, psychotic and depressive conditions co-aggregate. Cognitive impairment and major depressive disorder, a comorbidity widely assumed to be common in HIV, showed no significant association in this cohort once cognitive impairment was defined on strictly organic clinical grounds. A latent multimorbid chronic phenotype, independently recovered by two distinct methods, warrants prospective characterisation as a target for cognitive screening and integrated mental health care in ageing HIV populations. Precise, clinically grounded classification of ICD-10 diagnoses is recommended as a standard step for future HIV brain health research using routine clinical data.

### Supplementary Material

Supplementary Files

This is a list of supplementary files associated with this preprint. Click to download.

- Appendix.docx

## Figures and Tables

**Figure 2:**
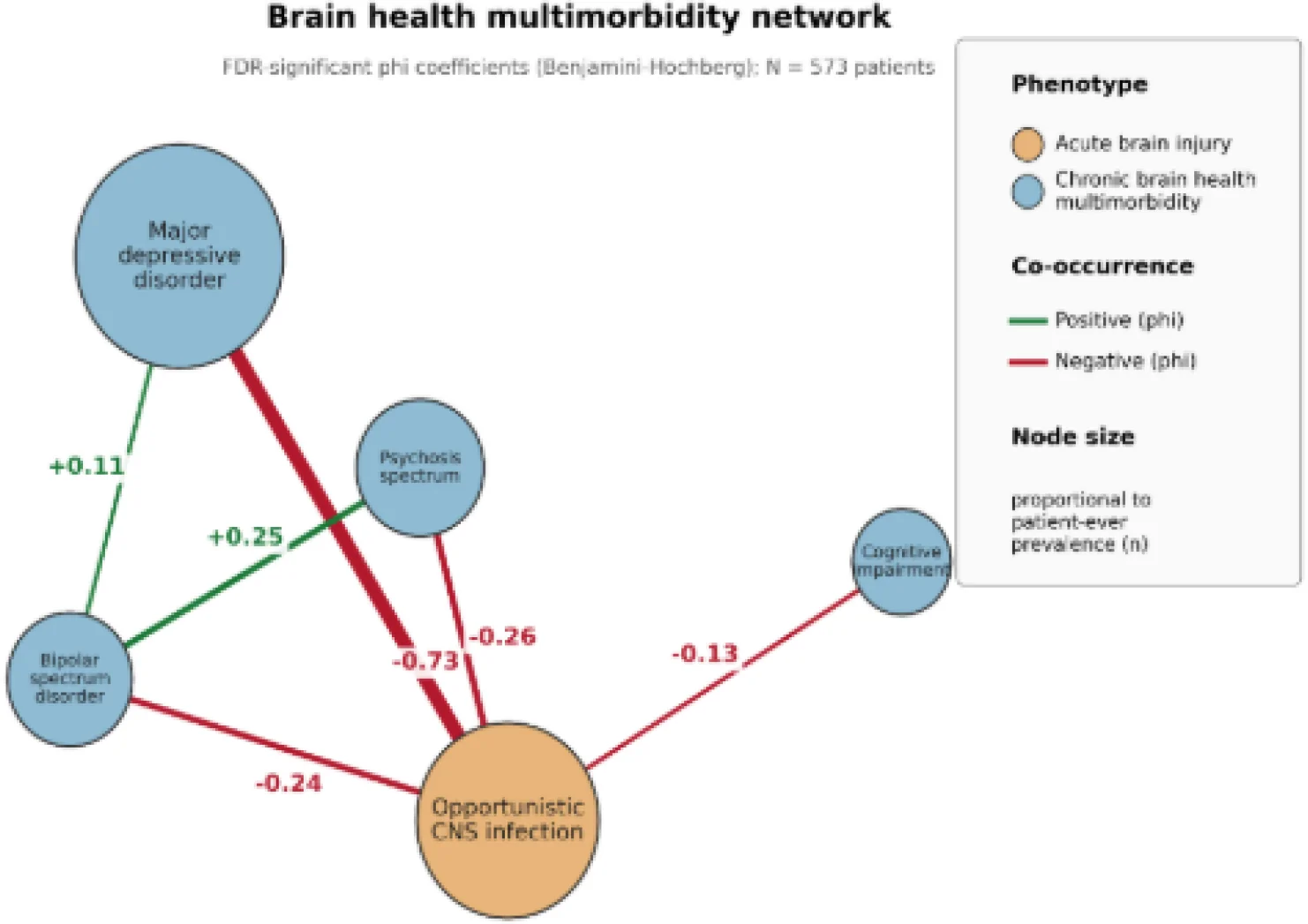
Brain health multimorbidity network among 573 classifiable people living with HIV. Nodes represent brain health domains, sized proportionally to patient-ever prevalence. Edges represent associations significant after Benjamini-Hochberg correction: green indicates positive co-occurrence and red indicates mutual exclusion. Orange nodes denote the acute brain injury phenotype; blue nodes denote the chronic brain health multimorbidity phenotype. Only the six significant associations (of ten tested) are shown.

**Figure 3:**
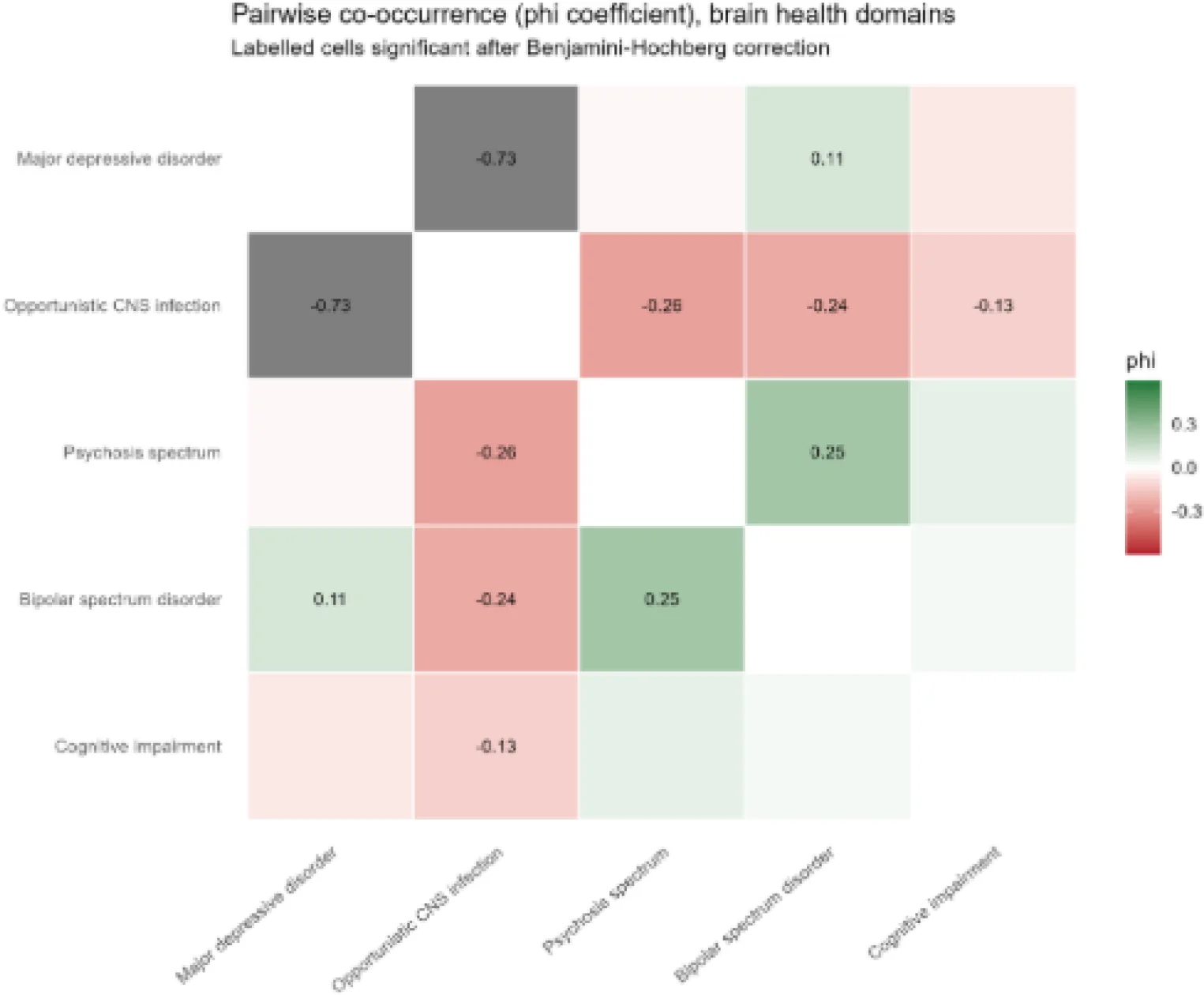
Pairwise co-occurrence heatmap of phi coefficients for all ten tested associations among the five brain health domains meeting the minimum cell-size threshold. Labelled cells are significant after Benjamini-Hochberg correction at the 0.05 false discovery rate threshold. The colour scale ranges from red (mutual exclusion) through white (independence) to green (co-aggregation).

**Table 1.** Pairwise co-occurrence of brain health domains.

Pair	Obs	Exp	O/E	Phi (95% CI)	q
Psychosis spectrum + Bipolar spectrum disorder	25	9.2	2.73	0.250 (0.144 to 0.366)	< 0.001
Major depressive disorder + Bipolar spectrum disorder	47	36.4	1.29	0.113 (0.033 to 0.186)	0.012
Opportunistic CNS infection + Cognitive impairment	3	11.4	0.26	−0.134 (−0.185 to −0.074)	0.002
Opportunistic CNS infection + Bipolar spectrum disorder	4	25.7	0.16	−0.240 (−0.288 to −0.189)	< 0.001
Opportunistic CNS infection + Psychosis spectrum	3	27.5	0.11	−0.263 (−0.310 to −0.210)	< 0.001
Major depressive disorder + Opportunistic CNS infection	8	109.2	0.07	−0.734 (−0.783 to −0.682)	< 0.001

Phi coefficients with 2,000-replicate bootstrap 95% confidence intervals. Only the six associations significant after Benjamini-Hochberg correction (of ten tested) are shown; the four non-significant associations, including cognitive impairment with major depressive disorder (phi −0.064, q = 0.203), are described in the text. Obs, observed joint count; Exp, expected under independence; O/E, observed/expected ratio; CI, confidence interval; q, false discovery rate-adjusted p value.

**Table 2.** Community assignments from signed spin-glass community detection (primary) and positive-edge-only Louvain community detection (sensitivity comparator).

Brain health domain	Signed community (primary)	Positive-edge Louvain (comparator)
Major depressive disorder	1 (Chronic cluster)	1 (Chronic cluster)
Bipolar spectrum disorder	1 (Chronic cluster)	1 (Chronic cluster)
Psychosis spectrum	1 (Chronic cluster)	1 (Chronic cluster)
Cognitive impairment	1 (via shared exclusion from acute phenotype)	3 (Isolate)
Opportunistic CNS infection	2 (Acute phenotype, isolate)	2 (Acute phenotype, isolate)

**Table 3.** Latent class analysis fit statistics.

Classes	Log-likelihood	AIC	BIC	Entropy	Smallest class (%)
1	−1328.9	2667.7	2689.5	n/a	100
2	−1083.2	2188.5	2236.3	0.972	35.2
3 (preferred)	−1032.2	2098.4	2172.4	0.856	24.6
4	−1018.5	2083.0	2183.1	0.904	2.8
5	−1012.6	2083.2	2209.4	0.927	1.9

The three-class model was preferred on the Bayesian information criterion (BIC). AIC, Akaike information criterion.

**Table 4.** Conditional probabilities of domain membership by latent class.

Brain health domain	Class 1: Depression (39.6%)	Class 2: Opportunistic infection (35.8%)	Class 3: Multimorbid (24.6%)
Major depressive disorder	1.000	0.026	0.467
Opportunistic CNS infection	0.000	1.000	0.033
Psychosis spectrum	0.005	0.000	0.524
Bipolar spectrum disorder	0.093	0.008	0.335
Cognitive impairment	0.008	0.004	0.201

BIC-preferred three-class model. Values above 0.30 indicate meaningful loading on the class.

## Data Availability

The datasets generated and analysed during the current study are not publicly available because they contain sensitive clinical information from an HIV care population, but the de-identified analytic dataset and the complete classification scheme are available from the corresponding author on reasonable request and with the permission of Mildmay Uganda. The analysis code is available from the corresponding author on reasonable request.

## Abbreviations

ART: antiretroviral therapy
BIC: Bayesian information criterion
CI: confidence interval
CNS: central nervous system
FDR: false discovery rate
HAND: HIV-associated neurocognitive disorder
HIV: human immunodeficiency virus
ICD-10: International Classification of Diseases, Tenth Revision
IQR: interquartile range
RECORD: REporting of studies Conducted using Observational Routinely-collected Data
STROBE: Strengthening the Reporting of Observational Studies in Epidemiology
VL: viral load

## References

[1] Rajasingham R, Govender NP, Jordan A, Loyse A, Shroufi A, Denning DW, The global burden of HIV-associated cryptococcal infection in adults in 2020: a modelling analysis. Lancet Infect Dis. 2022;22(12):1748–55. 10.1016/S1473-3099(22)00499-6. 36049486 PMC9701154

[2] Saylor D, Dickens AM, Sacktor N, Haughey N, Slusher B, Pletnikov M, HIV-associated neurocognitive disorder - pathogenesis and prospects for treatment. Nat Rev Neurol. 2016;12(4):234–48. 10.1038/nrneurol.2016.27. 26965674 PMC4937456

[3] Mekuriaw B, Belayneh Z, Teshome W, Akalu Y. Prevalence and variability of HIV/AIDS-associated neurocognitive impairments in Africa: a systematic review and meta-analysis. BMC Public Health. 2023;23(1):997. 10.1186/s12889-023-15935-x. 37254121 PMC10228136

[4] Nakku J, Kinyanda E, Hoskins S. Prevalence and factors associated with probable HIV dementia in an African population: a cross-sectional study of an HIV/AIDS clinic population. BMC Psychiatry. 2013;13:126. 10.1186/1471-244X-13-126. 23641703 PMC3653745

[5] Bernard C, Dabis F, de Rekeneire N. Prevalence and factors associated with depression in people living with HIV in sub-Saharan Africa: a systematic review and meta-analysis. PLoS ONE. 2017;12(8):e0181960. 10.1371/journal.pone.0181960. 28783739 PMC5544236

[6] Molapo DM, Mokgalaboni K, Phoswa WN. Prevalence of depression among people living with HIV on antiretroviral therapy in Africa: a systematic review and meta-analysis. Healthc (Basel). 2025;13(1):85. 10.3390/healthcare13010085. PMC11719924 39791692

[7] Petrushkin H, Boardman J, Ovuga E. Psychiatric disorders in HIV-positive individuals in urban Uganda. Psychiatr Bull. 2005;29(12):455–8. 10.1192/pb.29.12.455.

[8] Antinori A, Arendt G, Becker JT, Brew BJ, Byrd DA, Cherner M, Updated research nosology for HIV-associated neurocognitive disorders. Neurology. 2007;69(18):1789–99. 10.1212/01.WNL.0000287431.88658.8b. 17914061 PMC4472366

[9] Nightingale S, Dreyer AJ, Saylor D, Gisslen M, Winston A, Joska JA. Moving on from HAND: why we need new criteria for cognitive impairment in persons living with human immunodeficiency virus and a proposed way forward. Clin Infect Dis. 2021;73(6):1113–8. 10.1093/cid/ciab366. 33904889

[10] Benjamini Y, Hochberg Y. Controlling the false discovery rate: a practical and powerful approach to multiple testing. J R Stat Soc Ser B Stat Methodol. 1995;57(1):289–300. 10.1111/j.2517-6161.1995.tb02031.x.

[11] Reichardt J, Bornholdt S. Statistical mechanics of community detection. Phys Rev E. 2006;74(1):016110. 10.1103/PhysRevE.74.016110. 16907154

[12] Blondel VD, Guillaume JL, Lambiotte R, Lefebvre E. Fast unfolding of communities in large networks. J Stat Mech Theory Exp. 2008;2008(10):P10008. 10.1088/1742-5468/2008/10/P10008.

[13] Craddock N, Owen MJ. The beginning of the end for the Kraepelinian dichotomy. Br J Psychiatry. 2005;186(5):364–6. 10.1192/bjp.186.5.364. 15863738

[14] Sukumaran L, Winston A, Post FA, Anderson J, Boffito M, Sachikonye M, Changes in multimorbidity burden and their impact on patient and healthcare outcomes in people with HIV over a 3–5-year period. AIDS. 2025;39(12):1784–93. 10.1097/QAD.0000000000004260. 40478922 PMC12404628

[15] Tang B, Ellis RJ, Vaida F, Umlauf A, Franklin DR, Dastgheyb R, Biopsychosocial phenotypes in people with HIV in the CHARTER cohort. Brain Commun. 2024;6(4):fcae224. 10.1093/braincomms/fcae224. 39077377 PMC11285184

